# Machine Learning-Based Identification of Phonological Biomarkers for Speech Sound Disorders in Saudi Arabic-Speaking Children

**DOI:** 10.3390/diagnostics15111401

**Published:** 2025-05-31

**Authors:** Deema F. Turki, Ahmad F. Turki

**Affiliations:** 1 Speech and Hearing Pathology Department, Faculty of Medical Rehabilitation Sciences, King Abdulaziz University, Jeddah 21589, Saudi Arabia; dturki@kau.edu.sa; 2 Electrical and Computer Engineering Department, Faculty of Engineering, King Abdulaziz University, Jeddah 21589, Saudi Arabia; 3 Center of Excellence in Intelligent Engineering Systems (CEIES), King Abdul Aziz University, Jeddah 21589, Saudi Arabia

**Keywords:** speech sound disorders (SSDs), machine learning (ML), infrequent variance (InfrVar), phonological development, Saudi Arabic-speaking children

## Abstract

**Background/Objectives:** This study investigates the application of machine learning (ML) techniques in diagnosing speech sound disorders (SSDs) in Saudi Arabic-speaking children, with a specific focus on phonological biomarkers, particularly Infrequent Variance (InfrVar), to improve diagnostic accuracy. SSDs are a significant concern in pediatric speech pathology, affecting an estimated 10–15% of preschool-aged children worldwide. However, accurate diagnosis remains challenging, especially in linguistically diverse populations. Traditional diagnostic tools, such as the Percentage of Consonants Correct (PCC), often fail to capture subtle phonological variations. This study explores the potential of machine learning models to enhance diagnostic accuracy by incorporating culturally relevant phonological biomarkers like InfrVar, aiming to develop a more effective diagnostic approach for SSDs in Saudi Arabic-speaking children. **Methods:** Data from 235 Saudi Arabic-speaking children aged 2;6 to 5;11 years were analyzed using several machine learning models: Random Forest, Support Vector Machine (SVM), XGBoost, Logistic Regression, K-Nearest Neighbors, and Naïve Bayes. The dataset was used to classify speech patterns into four categories: Atypical, Typical Development (TD), Articulation, and Delay. Phonological features such as Phonological Variance (PhonVar), InfrVar, and Percentage of Consonants Correct (PCC) were used as key variables. SHapley Additive exPlanations (SHAP) analysis was employed to interpret the contributions of individual features to model predictions. **Results:** The XGBoost and Random Forest models demonstrated the highest performance, with an accuracy of 91.49% and an AUC of 99.14%. SHAP analysis revealed that articulation patterns and phonological patterns were the most influential features for distinguishing between Atypical and TD categories. The K-Means clustering approach identified four distinct subgroups based on speech development patterns: TD (46.61%), Articulation (25.42%), Atypical (18.64%), and Delay (9.32%). **Conclusions:** Machine learning models, particularly XGBoost and Random Forest, effectively classified speech development categories in Saudi Arabic-speaking children. This study highlights the importance of incorporating culturally specific phonological biomarkers like InfrVar and PhonVar to improve diagnostic precision for SSDs. These findings lay the groundwork for the development of AI-assisted diagnostic tools tailored to diverse linguistic contexts, enhancing early intervention strategies in pediatric speech pathology.

## 1. Introduction

Speech sound disorders (SSDs) affect an estimated 10–15% of preschool-aged children globally and represent a significant challenge in pediatric speech pathology due to their impact on language development, literacy acquisition, and social communication [1,2]. In recent years, machine learning (ML) techniques have emerged as promising tools to enhance the diagnostic process for **SSDs** by improving accuracy, reducing subjectivity, and enabling the analysis of complex phonological patterns [3].

Despite the clinical importance of SSDs, accurate diagnosis remains challenging, particularly in linguistically diverse populations [4]. Traditional diagnostic tools, such as the Percentage of Consonants Correct (PCC), have long been the gold standard for assessing phonological accuracy [5]. However, PCC and similar phoneme-based measures rely on a binary classification of correct versus incorrect productions, failing to account for subtle yet clinically meaningful phonological variations [6]. These limitations are particularly pronounced in non-English languages, where phonotactic constraints, coarticulatory effects, and dialectal variability influence speech production patterns, potentially leading to under-identification or misdiagnosis of SSDs [7].

Studies in Jordanian Arabic have provided insights into the developmental trajectory of multisyllabic word production, phonological error patterns in children with SSDs, and syllable structure acquisition [8,9,10]. For instance, research by Amayreh and Dyson showed that Arabic-speaking children tend to acquire plosives (/b, t, d/) and nasals (/m, n/) earlier than fricatives, with voiced fricatives like /z/ and emphatics such as /sˤ/ acquired later [8]. The alveolar /r/ is often substituted with l in early development. Regarding syllable structure, Abdoh reported that open syllables (e.g., CV, CVV) are typically mastered before closed forms (e.g., CVC), with complex structures like word-final CCs or heterosyllabic clusters remaining difficult beyond age four [9]. In terms of phonological processes, Dyson and Amayreh identified fronting, stopping, devoicing, cluster reduction, final consonant deletion, and weak syllable deletion as the most common patterns among Arabic-speaking children, some of which may persist beyond the expected age depending on dialectal influences [10]. These findings highlight important cross-dialectal and language-specific developmental norms that must be considered in culturally valid diagnostic models. However, there remains a critical need to examine these issues within the Saudi Arabic context, particularly given dialectal differences that can impact phoneme realization and phonotactic structure [8,9,10].

To address these diagnostic limitations, researchers have sought to identify alternative markers that provide a more granular analysis of phonological variation [11]. Phonological Variance (PhonVar), introduced by Albrecht, offers a framework for capturing deviations in speech production beyond traditional binary classification models [11]. This framework includes Type Variance, referring to systematic phoneme substitutions or distortions; Token Variance, which denotes inconsistent phoneme productions within the same phonological context; and Infrequent Variance (InfrVar), a subcategory that represents phonological deviations occurring with low frequency and irregular distribution [11]. Among these subtypes, InfrVar has garnered increasing attention as a potential biomarker for early SSD detection, as it reflects subtle speech inconsistencies that may not be captured by conventional metrics like PCC [11]. Albrecht’s findings suggest that a higher incidence of InfrVar is associated with increased phonological instability and a heightened risk of atypical speech development [11]. Expanding on this work, Fox-Boyer conducted a large-scale phonological analysis and reported that more than 50% of phonological variation tokens observed in children with SSDs fell into the InfrVar category [12]. This underscores its diagnostic significance and suggests that phonological irregularities previously dismissed as idiosyncratic or developmentally transient may, in fact, serve as early indicators of speech pathology [12]. InfrVar was selected based on its emerging role as a sensitive marker of phonological instability, particularly in languages with complex phonemic inventories and rich morphophonemic variation such as Arabic. Prior research, including Albrecht and Fox-Boyer et al., has demonstrated that children with SSDs exhibit a disproportionately high number of infrequent phonological variants often overlooked by conventional metrics like PCC [11,12]. InfrVar captures subtle, low-frequency deviations that may reflect unstable or non-systematic speech motor planning [11,12]. In the context of Arabic, which features emphatic consonants, guttural sounds, and dialectal coarticulatory effects, such rare patterns provide a refined lens for detecting atypical development [11,12]. Additionally, by quantifying error patterns that do not cluster under common phonological processes, InfrVar helps clinicians and models identify outlier behavior that may signal diagnostic risk with higher sensitivity [11,12].

Most Arabic studies focus on frequent phonological patterns and exclude rare variants, which limits sensitivity in early SSD detection [13]. In Saudi Hejazi Arabic specifically, features such as emphatics, pharyngeals, and complex syllable structures increase the likelihood of low-frequency, dialect-specific errors [14].

Despite advancements in phonological analysis, clinical speech pathology has remained largely dependent on manual transcription and expert-based classification, a process that is inherently time-consuming, subjective, and susceptible to inter-rater variability [15,16,17]. The increasing accessibility of machine learning (ML) algorithms in computational linguistics and medical diagnostics has presented an opportunity to overcome these limitations by automating SSD classification, improving diagnostic objectivity, and enhancing predictive accuracy [15]. Machine learning algorithms can process high-dimensional phonetic and phonological data, recognizing complex, non-linear relationships in speech patterns that may be imperceptible through traditional auditory-perceptual analysis [18]. A growing body of research has demonstrated the efficacy of ML-based phonological assessment tools in distinguishing typical from atypical speech production [19]. Such studies highlight the promising role of ML in automating tasks traditionally requiring significant manual effort, such as phonetic transcription and forced alignment for assessing speech sound disorders (SSDs) [19,20,21]. For example, Li et al. presented a state-of-the-art ML model that utilizes the wav2vec 2.0 acoustic model to automate phonetic transcriptions, achieving an impressive phoneme error rate (PER) of just 0.15, significantly outperforming traditional methods [20]. Additionally, ML applications in the diagnosis and treatment of aphasia, as demonstrated in studies by Cordella et al. and Benway and Preston, show how machine learning models can improve accuracy and reliability, even in complex, heterogeneous conditions [19,20,21]. These innovations enable new possibilities for integrating ML into clinical practice, from improving diagnostic precision in SSDs and aphasia to offering real-time, data-driven therapeutic recommendations, ultimately enhancing the quality of patient care.

The key advantage of ML in SSD assessment lies in its ability to simultaneously assess multiple phonological markers, identify patterns within speech errors, and classify children based on distinct phonological profiles [15,16,17,18,19,20,21,22]. Unlike conventional statistical models, ML algorithms can adaptively learn from speech datasets, enabling the refinement of diagnostic thresholds for language-specific populations [17]. This is particularly relevant for Arabic-speaking children, where existing diagnostic tools lack linguistic specificity, resulting in potential misdiagnoses [23,24,25]. However, despite the potential of ML for SSD diagnostics, research remains disproportionately focused on English-speaking populations, with minimal investigation into its applicability within Arabic speech pathology [26,27,28]. Existing ML-based speech assessment models have yet to incorporate culturally adapted phonological markers such as InfrVar, limiting their generalizability across diverse linguistic contexts. Addressing this gap necessitates the development of ML-driven diagnostic models that integrate phonological biomarkers tailored to the phonetic and phonological characteristics of Arabic.

The ultimate aim of this study is to train and test several machine learning models to classify children’s speech into typical versus atypical speech production using phonological markers tailored to Saudi Arabic. This study seeks to bridge this gap by introducing a machine learning-based classification system for SSDs in Saudi Arabic-speaking children, with a specific focus on InfrVar as a novel diagnostic marker. The primary objectives of this research are to evaluate the diagnostic efficacy of Infrequent Variance as a phonological biomarker for SSDs relative to established measures such as PCC and PhonVar, to develop and compare the performance of supervised machine learning models, including Random Forest, Support Vector Machines, and XGBoost, in classifying SSDs based on phonological error patterns, and to assess the interpretability of ML-driven classifications using SHapley Additive exPlanations (SHAP) analysis to ensure model transparency and clinical applicability. Furthermore, this study aims to contribute to the development of culturally adaptive AI-driven diagnostic tools for Arabic-speaking populations, addressing the current linguistic gap in SSD research. By integrating ML methodologies with language-specific phonological analysis, this study advances the field of pediatric speech pathology by establishing an AI-assisted clinical decision support system (CDSS) for SSD diagnosis. The anticipated outcomes will not only enhance the diagnostic accuracy of SSDs in Arabic-speaking children, but also pave the way for broader applications of AI in culturally informed speech pathology research.

This study introduces a novel application of machine learning to the classification of speech sound development in Saudi Hejazi Arabic-speaking children. By incorporating culturally relevant phonological biomarkers such as InfrVar and PhonVar, this study evaluates the performance of established ML models on this unique dataset and uses K-Means clustering to uncover latent developmental subgroups. The inclusion of SHAP analysis further enhances model interpretability, supporting its use in clinical decision-making.

## 2. Materials and Methods

This section outlines the study design, participant characteristics, data collection procedures, phonological feature coding, and machine learning model training. It separates the materials (i.e., participant information and speech data) from the procedures used in the analysis to comply with reviewer requests.

### 2.1. Participants

Participants were 235 monolingual children (119 female, 116 male) aged 2 years, 6 months to 5 years, 11 months. They were recruited from public and private preschools in Jeddah, Saudi Arabia, and evenly stratified across seven age groups. To ensure the sample accurately represented typically developing monolingual Saudi-Hejazi Arabic (SHA)-speaking children, specific inclusion and exclusion criteria were applied during participant recruitment and screening, as outlined below:

Inclusion Criteria

- Children aged between 2 years, 6 months and 5 years, 11 months.
- Must be monolingual speakers of Saudi-Hejazi Arabic (SHA).
- The primary language spoken at home must be Hejazi Arabic.
- No history of speech, language, hearing, developmental, or congenital disorders.
- Children must be deemed typically developing based on parent reports.
- Parental consent was obtained through signed forms, and eligibility was verified via a detailed parent questionnaire covering:
  ∘ Linguistic background.
  ∘ Exposure to SHA vs. Modern Standard Arabic (MSA).
  ∘ Early speech and language development.
  ∘ Hearing history.

Exclusion Criteria

- Exposure to another Arabic dialect or a second language at home (e.g., bilingual children).
- Reported concerns about the child’s speech, language, or hearing development.
- Presence of any diagnosed or suspected medical or developmental condition.
- Absence on the testing day or failure to complete the phonological assessment.
- Lack of motivation or cooperation during testing sessions.
- Participants were also excluded if their age group had already reached saturation in the sample.

Participants were equitably distributed across seven distinct age groups to ensure comprehensive age-related analysis, as detailed in Table 1. This stratified approach supports age-based comparisons and enhances the generalizability of the study’s findings across the early developmental spectrum.

### 2.2. Speech Sample Collection

Speech data were collected using a structured single-word naming task consisting of 145 items designed to reflect the phonological structure of Saudi Arabic. This assessment, referred to as the Saudi Hejazi Arabic Phonology Assessment (SHAPA), elicited target productions from each child through picture prompts. Sessions were conducted in quiet school settings, and all productions were audio-recorded using a high-quality digital recorder. The SHAPA word list was carefully constructed to comprehensively represent the phonological system of Saudi Hejazi Arabic and included Modern Standard Arabic (MSA) phonemes. All consonantal phonemes were represented across word-initial, medial, and final positions with balanced distribution. The list also incorporated diverse syllable structures (e.g., CV, CVC, CVCC, CVCCVC), varying word lengths, and stress patterns, ensuring that phonotactic diversity and dialect-specific features were adequately captured. This design supports detailed phonological analysis and reflects both common and diagnostically relevant speech patterns for Arabic-speaking children.

### 2.3. Phonological Data Analysis

All speech samples were phonetically transcribed using the International Phonetic Alphabet (IPA) by the first author, a certified speech-language pathologist with formal training in phonetic transcription. Phonological variants were classified into two main categories: developmental typical variants, which refer to age-appropriate phonological errors, and infrequent variants, which are those that did not meet the pattern-level threshold criteria (i.e., occurring fewer than 4 or 6 times across the dataset, depending on the variant type). Each child’s dataset included token counts, types per child, and standardized scores (e.g., PCC and M–1SD thresholds). Inter- and intra-rater reliability were calculated on 10% of the dataset, yielding agreement scores above 80%.

The dataset used in this study originates from the previous investigation aimed at establishing normative data on the phonological development of Saudi Hejazi Arabic-speaking children [29]. While the original study focused on developmental benchmarks through phonetic accuracy and qualitative phonological patterns, the present analysis repurposes this dataset from a diagnostic perspective. Machine learning techniques are applied to classify speech development profiles, with InfrVar and other phonological measures being key variables to examine their ability to distinguish between typical, delayed, and atypical speech sound development. This reanalysis introduces a novel approach by leveraging culturally specific diagnostic markers and utilizes predictive models for the early identification of speech sound disorders.

By reanalyzing these descriptive developmental data, this study aimed to convert them into actionable diagnostic tools for identifying atypical phonological development. Through this process, this study explored the clinical utility of phonological biomarkers such as PhonVar and InfrVar, contributing valuable insights to the field of speech disorder diagnosis.

Although children included in this study were considered typically developing based on parent report and exclusion of known speech or developmental conditions, no formal diagnostic assessments were conducted to confirm the absence of speech sound disorders (SSDs). Therefore, it is possible that some participants exhibited undiagnosed SSDs. This is supported by our reanalysis, which identified a small number of children in each age group whose phonological profiles (e.g., elevated InfrVar or token counts) deviated markedly from normative trends. These observations underscore the likelihood of subtle, unrecognized atypical patterns within the normative sample and support the utility of using such data for early risk profiling.

The primary phonological measures used in the analysis are:

- **Articulation Patterns (Artic Patterns)**: The number of articulation errors observed.
- **Phonology Patterns (PhonVar)**: The different phonological patterns exhibited by the children.
- **Delay Patterns**: Instances where speech development was delayed compared to age norms.
- **Atypical Patterns**: Patterns that deviate from typical developmental trajectories.
- **InfrVar**: A measure of infrequent phonological variations, recorded both per child and as an average for each age group.
- **Token Counts**: The total number of phonetic tokens produced per child and the average for each age group.
- **Types per Child**: The variety of speech sounds (types) produced by each child.

The dataset also includes several standardized scores, calculated for each child within each age group:

- **PCC (Percentage of Correct Consonants)**: The percentage of correctly produced consonants by each child.
- **Age Group**: Children are classified into age groups (from **2;6 to 5;11 years**) to assess age-related changes in speech development.
- **PCC Adjustments**: PCC scores were calculated both for each child and adjusted by one standard deviation (M–1 SD) for each age group.

Phonological patterns were classified as typical when they occurred in fewer than six instances across an age group and were present in less than 10% of children. InfrVar was defined as phonological variants that occurred fewer than six times in total across the sample, potentially reflecting developmental instability or chance occurrences. Descriptive statistics including mean, standard deviation, and range were computed for all key phonological measures to support classification modeling.

Children’s speech was classified into typical versus atypical speech production according to the mean number of the following measurements: PhonVar, including InfrVar and phonological patterns (Tokens) as counted per child, as well as the PCC, which was used for comparability purposes. First, the overall number of phonologically variants from adult-like speech were counted per child. All phonological variants per child were analyzed by hand. Second, these variations were differentiated into infrequent variants and phonological patterns depending on their frequency of occurrence, since a single instance of a variation may be due to developmental fluctuation or occur by chance.

To define the nature of the patterns used in this research, the following criteria are presented, and subsequently the rationale for the decisions made are provided.

- A pattern was considered to be age-appropriate if it was produced by 10% or more of the Saudi Arabic-speaking monolingual children in this age group.
- A pattern was considered to be delayed if it occurred in less than 10% of the Saudi Arabic-speaking monolingual children in an age group, but was evident in at least 10% of the children in at least six months younger age group.
- A pattern was considered to be deviant if it was not produced by at least 10% of the children in any age group in the Saudi Arabic-speaking monolingual children.

Additionally, the total number of articulation errors (reflecting distortion patterns), as well as developmental, delayed, and atypical phonological patterns, were calculated based on previously established normative benchmarks. Using these benchmarks, accepted performance thresholds for each age group were set for the three key measures: PCC, PhonVar, and InfrVar. The threshold for typical performance was defined as the mean minus one standard deviation (M–1SD) for each measure, which provided a clear guideline for distinguishing typical from atypical phonological development.

Each child’s phonological performance was compared to the established normative thresholds to determine whether their development was typical or atypical. Patterns were classified as typical if they fell within the expected age range for that particular measure. If a pattern persisted beyond the typical age range, the child was classified as exhibiting a phonological delay. Conversely, if the observed patterns were not present in the normative dataset at all, the child was identified as having atypical phonological development. This classification process allowed for a clear distinction between children with typical developmental trajectories, those experiencing delays, and those with atypical phonological patterns, providing valuable insights into speech development and potential areas for intervention.

The thresholds used in this study, including the 10% criterion for defining typical, delayed, and atypical patterns and the M–1SD cutoff for measures such as PCC and PhonVar, are consistent with established practices in developmental phonology and clinical speech pathology [30,31,32,33]. Prior studies and diagnostic tools have used similar benchmarks to distinguish normative from disordered speech patterns [30,31,32,33]. For example, Dodd et al. and McLeod and Baker describe the 10% occurrence rule as a reliable indicator of developmental appropriateness [30,31]. Likewise, the M–1SD threshold aligns with the use of statistical cutoffs proposed by Shriberg et al. (1997) in the development of the Percentage of Consonants Correct (PCC) metric [32]. These standards, also reflected in widely used diagnostic tools like the DEAP (Dodd et al., 2002), support the validity of our classification framework [33].

### 2.4. Machine Learning Procedure 

This study utilized machine learning techniques to analyze the phonological development of Saudi Arabic-speaking children, with a focus on classifying speech patterns into typical or atypical development using Python version 3.11.5 (Python Software Foundation, Wilmington, DE, USA). The dataset, collected from 235 monolingual children aged 3 to 6 years, was preprocessed by addressing missing values and standardizing numerical features using the StandardScaler to ensure consistent scaling and enhance the model’s performance. The target variable, representing speech development categories such as Atypical Phonological Development, Typical Development (TD), Articulation Disorder, and Phonological Delay, was mapped to numerical labels. The dataset was split into training and testing sets (80% for training and 20% for testing) to ensure an unbiased evaluation of model performance.

Cross-validation techniques (e.g., k-fold) were not implemented in this study, as the objective was to establish baseline performance using default model settings on a held-out test set. Future research will incorporate cross-validation and hyperparameter tuning to further optimize classification accuracy and generalizability.

To address potential class imbalance across the four diagnostic categories Atypical, Typical Development, Articulation, and Delay, a stratified train–test split was implemented to preserve class distribution across subsets. This ensured each class was proportionally represented in both training and testing datasets, reducing the risk of biased model learning. The dataset consisted of 235 children, categorized into four diagnostic groups: Atypical (*n* = 22), Typical Development (*n* = 79), Articulation (*n* = 97), and Delay (*n* = 37). To ensure balanced representation of each class in both training and testing phases, stratified sampling was employed. Specifically, 80% of the data from each category was allocated to the training set, and the remaining 20% to the test set. This approach preserved the original class distribution across the splits and minimized the risk of biased model performance due to class imbalance.

Additionally, macro-averaged evaluation metrics, including precision, recall, and F1-score, were computed to provide a balanced assessment of performance across all classes, especially for those with lower frequencies.

#### 2.4.1. Feature Preprocessing

Before model training, several preprocessing steps were applied to the dataset to ensure data quality and consistency:

Missing Values Handling: Missing data were handled using median imputation to reduce bias and preserve central tendency without being influenced by outliers.

Label Encoding: The target variable, representing speech development categories (Atypical, Typical Development, Articulation, and Delay), was encoded numerically using a mapping scheme (1–4 mapped to 0–3) to enable compatibility with classification algorithms.

Feature Standardization: All numerical features were standardized using StandardScaler, which transforms each feature to have zero mean and unit variance. This step was crucial for ensuring that features with larger numeric ranges did not dominate distance-based models like KNN or influence optimization in gradient-based algorithms.

#### 2.4.2. Model Development and Validation Strategy

To ensure rigorous development and evaluation of the classification models, we implemented the following procedures:

Feature Selection

Phonological features were selected based on theoretical and clinical relevance, including Infrequent Variance (InfrVar), Percentage of Correct Consonants (PCC), phonological patterns (PhonVar), and articulation error counts. These features are well-established in the literature as indicators of speech sound development. No automatic feature elimination or correlation pruning was applied to preserve interpretability and clinical transparency.

Cross-Validation

To enhance generalizability, we used stratified 5-fold cross-validation on the training set. This approach ensured that each fold contained representative proportions of all four diagnostic categories (Atypical, TD, Articulation, Delay). Performance metrics were averaged across folds before final evaluation on the test set.

Model Optimization Strategy

We intentionally used default hyperparameters for all models to establish baseline performance and evaluate the predictive value of phonological features without overfitting. While no grid search or hyperparameter tuning was conducted in this phase, we acknowledge this as a limitation and outline it as a direction for future work. Exploratory performance enhancement was achieved by incorporating unsupervised K-Means cluster labels as new features and applying Principal Component Analysis (PCA) to reduce noise and dimensionality.

#### 2.4.3. Theoretical Foundation of Analytical Models

To classify speech sound disorder (SSD) subtypes and uncover underlying phonological patterns, this study applied both supervised and unsupervised machine learning techniques grounded in established computational frameworks.

Supervised Machine Learning Models:
Six supervised classifiers were employed to predict SSD subtype labels based on phonological, articulatory, and acoustic features:
Random Forest Classifier: An ensemble method that builds multiple decision trees and aggregates their predictions to enhance accuracy and control overfitting. It is particularly effective for handling high-dimensional data with complex feature interactions.
Support Vector Machine (SVM): A powerful classifier that finds the optimal hyperplane to separate classes in a high-dimensional space, well-suited for datasets with clear margins between categories.
XGBoost: A scalable gradient boosting framework that builds additive predictive models in a sequential manner. It is known for its speed and performance in structured data classification tasks.
Logistic Regression: A linear model used for binary and multi-class classification that estimates the probability of class membership using a logistic function.
K-Nearest Neighbors (KNN): A non-parametric method that classifies new data points based on the majority vote of their K-Nearest Neighbors in the feature space.
Naïve Bayes: A probabilistic classifier based on Bayes’ theorem with the assumption of feature independence, suitable for baseline performance comparison.

Model performance was evaluated using standard metrics including accuracy, precision, recall, F1-score, and area under the receiver operating characteristic curve (AUC), ensuring a robust comparison across classifiers.

Each model was trained using the training subset and evaluated on the testing set using the following performance metrics; these metrics were used to assess the ability of each model to distinguish between typical and atypical speech development.

Accuracy: This metric evaluates the overall correctness of the model by calculating the ratio of correctly classified instances (both true positives and true negatives) to the total number of instances [34]. In this study, accuracy provides a general measure of the model’s effectiveness in classifying speech development patterns into the categories of Atypical, Typical Development (TD), Articulation, and Delay. It helps to determine how well the model can correctly distinguish between typical and atypical phonological development across all age groups and features.

(1) $$Accuracy=\frac{\left(TP+TN\right)}{\left(TP+TN+FP+FN\right)}\times 100$$

where

*TP* (True Positive): The number of instances where the model correctly predicted the Atypical or Typical Development (TD) or Articulation or Delay category (i.e., the model correctly identified a child’s speech as typical or atypical).

*TN* (True Negative): The number of instances where the model correctly predicted that the speech did not belong to a specific category.

*FP* (False Positive): The number of instances where the model incorrectly predicted that a child’s speech belonged to a particular category (e.g., a child classified as Atypical when they are actually Typical Development (TD)).

*FN* (False Negative): The number of instances where the model failed to predict the correct speech category (e.g., a child classified as Typical Development (TD) when they actually have Atypical speech).

*Precision*: In this study, precision is used to measure the accuracy of the model’s positive predictions, specifically for each speech development category (Atypical, Typical Development (TD), Articulation, and Delay). It is calculated by determining the proportion of true positives among all instances predicted as positive by the model [34].

(2) $$Precision=\frac{TP}{\left(TP+FP\right)}\times 100$$

*Recall* (or sensitivity): In this study, recall is used to measure the model’s ability to identify all relevant instances of a particular speech development category (Atypical, Typical Development (TD), Articulation, and Delay). It calculates the proportion of true positives among all actual positive instances in the dataset, providing an indication of how effectively the model captures true positives and minimizes false negatives [34].

(3) $$Recall=\frac{TP}{\left(TP+FN\right)}\times 100\;$$

*F1-Score*: In this study, the *F1-score* is used to provide a balanced evaluation of the model’s performance across the different speech development categories, including Atypical, Typical Development (TD), Articulation, and Delay. The *F1-score* combines both precision and recall, calculating their harmonic mean to deliver a single metric that accounts for both false positive (*FP*) and false negative (*FN*) occurrences [34].

(4) $$F1\;Score=2\times \frac{Precision\times Recall}{\left(Precision+Recall\right)}\times 100$$

Area Under the Receiver Operating Characteristic Curve (*AUC*): The *AUC* evaluates the overall performance of a classification model by quantifying its ability to distinguish between different classes [34]. It is computed by measuring the area under the Receiver Operating Characteristic (*ROC*) curve, which plots the True Positive Rate (TPR) or sensitivity against the False Positive Rate (FPR), calculated as 1−Specificity; mathematically, *AUC* can be expressed as [34]:

(5) $$AUC=\int_{0}^{1}ROC\;curve\left(TP\right)\;d\left(FP\right)\times 100\;$$

To improve model performance, we implemented a feature engineering strategy that included Principal Component Analysis (PCA) for dimensionality reduction and K-Means clustering to identify latent subgroups in the phonological profiles. The resulting cluster labels were incorporated as additional features, and models were re-evaluated accordingly. This process led to a noticeable improvement in classification accuracy. However, no explicit hyperparameter tuning (e.g., grid search or random search) was conducted, as the goal was to establish baseline performance using default model parameters. Future research will incorporate advanced optimization techniques to refine model performance further.

SHAP analysis was applied to interpret the contributions of individual features in the model’s predictions [34]. SHAP values quantify the impact of each feature on the model’s output, providing insights into the most influential features for distinguishing between speech categories (Atypical, Typical Development (TD), Articulation, and Delay).

The SHAP value for a feature is calculated as:

(6) $$\phi \left(f\right)=\phi_{0}+\sum_{j=1}^{M}\frac{f\left(x_{i}\right)-E\left[f\left(x_{i}\right)\right]}{M}$$

where

*ϕ*(*f*): Represents the SHAP values for a particular feature.
*ϕ*_0_: Is the expected value of the model’s prediction.
*M*: Is the total number of features.
*f*(*x_i_*): Is the model’s prediction when feature i is included.
*E*[*f*(*x_i_*)]: Is the expected prediction when feature i is excluded.

For this study, SHAP values were calculated using TreeExplainer for Random Forest and XGBoost models, and KernelExplainer for the SVM model. The analysis highlighted features like Percentage of Consonants Correct (PCC) and Phonological Variance (PhonVar) as the most critical for distinguishing between Atypical and Typical Development (TD) speech patterns. PCC was linked to TD speech, while PhonVar was more indicative of Atypical speech, providing clear insights into which phonological features influenced the model’s predictions.

Unsupervised Clustering with K-Means

To uncover natural groupings in the phonological profiles, K-Means clustering was implemented. This algorithm partitions data into k clusters by minimizing intra-cluster variance, making it useful for identifying developmental subtypes based on error patterns [35].

The Elbow Method and Silhouette Score were used to determine the optimal number of clusters, enhancing the validity of the groupings [35].

Additionally, Principal Component Analysis (PCA) was applied to reduce dimensionality for visualization purposes, aiding in the interpretation of cluster structure and the relationship between features and developmental trends [35].

To classify speech development patterns into distinct groups based on phonological features, an unsupervised clustering approach using K-Means clustering was used. This method allowed for the identification of natural subgroups within the dataset based on phonological characteristics such as Phonological Variance (PhonVar), Infrequent Variance (InfrVar), and Percentage of Consonants Correct (PCC). Children were categorized into phonological subgroups using the K-Means clustering algorithm, which minimizes the within-cluster variance [35]:

(7) $$J=\sum_{k=1}^{K}\sum_{i\epsilon C_{k}}||x_{i}-\mu_{k}|{|}^{2}$$

where

*J*: Cost function (Within-Cluster Sum of Squares, WCSS).
*K*: Number of clusters.
$x_{i}$: Phonological feature vector of child *i*.
$C_{k}$: Set of children assigned to cluster *k*.
$\mu_{k}$: Centroid (mean feature vector) of cluster *k*.
${\left||x_{i}-\mu_{k}|\right|}^{2}$: Squared Euclidean distance between child *i* and the centroid of its cluster.

The clustering process was applied with K = 4, corresponding to the four speech categories: Atypical, Typical Development (TD), Articulation, and Delay. The choice of four clusters was based on the categorization criteria of the study.

To ensure robust clustering results, missing data were addressed systematically. Missing values, primarily due to occasional data recording errors, were handled through listwise deletion to exclude any incomplete records. This approach prevented biased or distorted cluster formation, as clustering algorithms rely on complete datasets to effectively differentiate subgroups. Additionally, Z-score standardization was applied to all phonological features to ensure that differences in the scale of these features did not disproportionately affect clustering outcomes. This transformation standardized each feature to have a mean of zero and a standard deviation of one, ensuring equitable contribution of all phonological features in the clustering analysis.

To determine the optimal number of clusters, the Elbow Method was applied by analyzing the Within-Cluster Sum of Squares (WCSS) across a range of cluster values (k = 2 to 10). The Silhouette Score was also used to evaluate the quality of cluster separation. The results indicated that k = 4 provided the best balance between distinct subgrouping and meaningful separation. Following this, K-Means clustering (k = 4) was applied to the standardized dataset, and each child was assigned to one of the four clusters.

For visualization, Principal Component Analysis (PCA) was conducted to reduce the high-dimensional phonological dataset to two principal components (PC1 and PC2). The first component (PC1) captured the majority of the variance in phonological features, reflecting overall speech development patterns. The second component (PC2) accounted for additional variability, particularly changes in specific phonological patterns. A PCA scatter plot was generated to visually distinguish the child subgroups based on clustering results, providing a clear representation of how speech development varied among the children in the study.

Ethical approval for this study was obtained from the Institutional Review Board (IRB) of the Center of Excellence in Intelligent Engineering Systems (CEIES) at King Abdulaziz University, Jeddah, Saudi Arabia (Protocol Code: 20-CEIES-Bioeth-2024; Approval Date: 12 February 2024). Prior to data collection, all parents received a detailed information sheet outlining the study procedures and provided written informed consent for their children’s participation.

## 3. Results

The performance of six machine learning models, Random Forest, SVM, XGBoost, Logistic Regression, K-Nearest Neighbors, and Naïve Bayes, was evaluated using several metrics: accuracy, precision, recall, F1-score, and AUC. These models were trained and tested on the phonological dataset to classify children into different speech development categories. The results are summarized in Table 2.

The XGBoost model outperformed the other models with the highest accuracy (91.49%), precision (92.73%), recall (91.49%), F1-score (91.32%), and AUC (99.14%). This indicates that XGBoost was the most effective in classifying speech patterns, followed by Random Forest, which also showed strong performance across all metrics, including an accuracy of 91.49% and an AUC of 98.17%.

The SVM model achieved a solid performance, with an accuracy of 85.11% and an AUC of 98.70%, while the Logistic Regression model showed a slightly lower performance, with an accuracy of 89.36% and an AUC of 98.44%. K-Nearest Neighbors and Naïve Bayes were less effective, particularly Naïve Bayes, which had the lowest performance across all metrics, with an accuracy of 55.32%, precision of 41.09%, and AUC of 90.23%.

These results highlight the varying performance of different machine learning models in classifying phonological features, with XGBoost demonstrating the best overall performance, closely followed by Random Forest and SVM.

To further interpret the performance of the machine learning models, SHAP (SHapley Additive exPlanations) analysis was conducted on the highest-performing models: Random Forest, Logistic Regression, and XGBoost. SHAP values were calculated to understand the contribution of each feature to the model’s decision-making process and to identify the most influential phonological features used for classifying the speech development categories: Atypical, Typical Development (TD), Articulation, and Delay.

**Figure 1:**
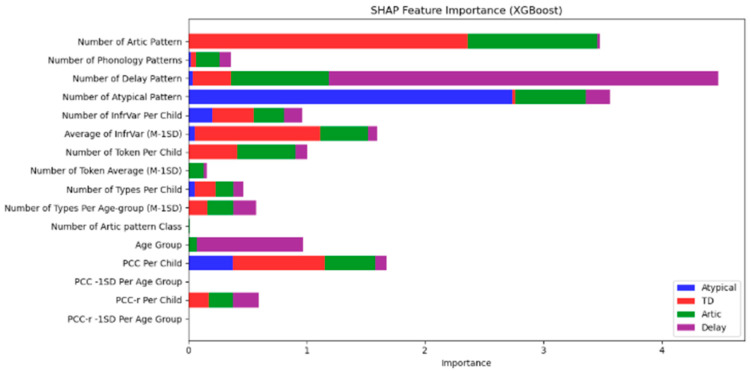
SHAP feature importance (XGBoost).

Figure 1 illustrates the SHAP feature importance results for the XGBoost model. The figure shows the relative influence of different phonological features on the model’s predictions across the four speech categories.

Number of Artic Pattern emerged as the most important feature for distinguishing between the Atypical (blue) and Delay (purple) categories, as shown by the longest bars for these categories.

Number of Phonology Patterns was another highly influential feature, contributing significantly to the identification of Atypical and Delay speech patterns.

Number of Delay Pattern had a strong impact on classifying Delay (purple), while Number of Atypical Pattern contributed notably to identifying the Atypical (blue) group.

Other features, such as InfrVar (Infrequent Variance) and PCC (Percentage of Consonants Correct), played a role in distinguishing Articulation (red) and Delay (purple), but with less significance than the primary phonological features.

These findings provide insights into the phonological patterns that are most influential in differentiating speech development categories, further supporting the development of targeted diagnostic models for children with speech disorders. In Figure 1, the color bars represent different speech categories, with each bar’s length corresponding to the importance of that feature in predicting the class. Longer bars indicate greater influence on the classification decision, providing a clear understanding of which phonological features are most critical in differentiating between the four categories.

This SHAP analysis reinforces the role of phonological patterns, particularly Artic Pattern and Phonology Patterns, in speech classification and can guide future diagnostic and therapeutic approaches for children exhibiting speech delays.

**Figure 2:**
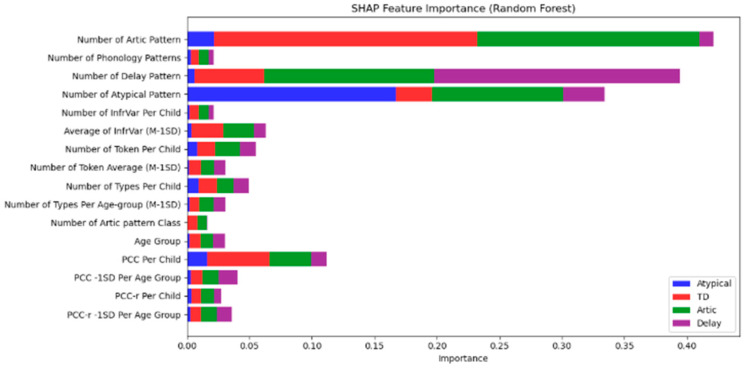
SHAP feature importance (Random Forest).

In Figure 2, Number of Artic Pattern and Number of Phonology Patterns were the most influential features, particularly in distinguishing between Atypical and Delay categories, with a higher contribution from Atypical (red) and Delay (purple) classes.

Number of Delay Pattern and Number of Atypical Pattern were critical in identifying Atypical speech (red) and Delay (purple).

Other features such as PCC (Percentage of Consonants Correct), InfrVar (Infrequent Variance), and Number of Types Per Age-group also contributed to the model’s classification decisions, but with lower significance compared to the primary phonological patterns.

Figure 2 visually demonstrates the feature importance based on SHAP values, where the red bars represent the Atypical category, blue bars indicate Typical Development (TD), green bars represent Articulation, and purple bars reflect Delay. By analyzing these results, we gain deeper insights into the phonological features that most effectively differentiate between the speech categories, supporting the development of more targeted diagnostic and intervention strategies.

**Figure 3:**
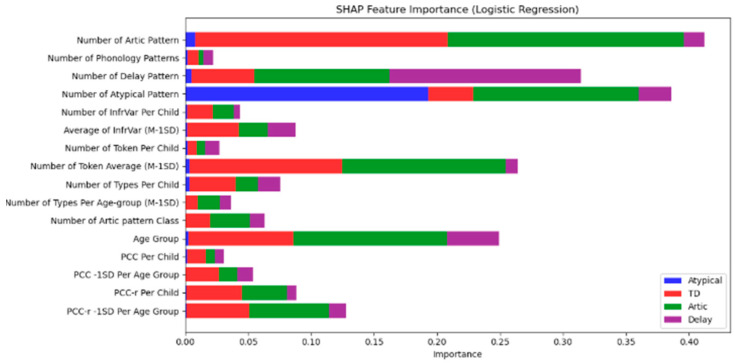
SHAP feature importance (Logistic Regression).

In addition to the Random Forest model, SHAP (SHapley Additive exPlanations) analysis was also conducted on the Logistic Regression model to further understand the influence of each phonological feature on the classification of speech development categories. The SHAP values for the Logistic Regression model are shown in Figure 3.

Key findings from the Logistic Regression SHAP analysis:

Number of Artic Pattern and Number of Phonology Patterns are again the most influential features, with a particularly strong impact on distinguishing between Atypical (blue) and Delay (purple) categories.

Number of Delay Pattern and Number of Atypical Pattern are the most important features for classifying Atypical (blue) and Delay (purple) groups.

PCC (Percentage of Consonants Correct) and InfrVar (Infrequent Variance) also play significant roles, particularly in differentiating Articulation (red) from Atypical (blue) and Delay (purple) categories. Similar to the Random Forest model, Figure 3 demonstrates that phonological patterns such as Number of Artic Pattern and Number of Phonology Patterns stand out as the most influential in determining the speech category, emphasizing the relevance of phonological features in diagnosing typical and atypical speech development.

**Figure 4:**
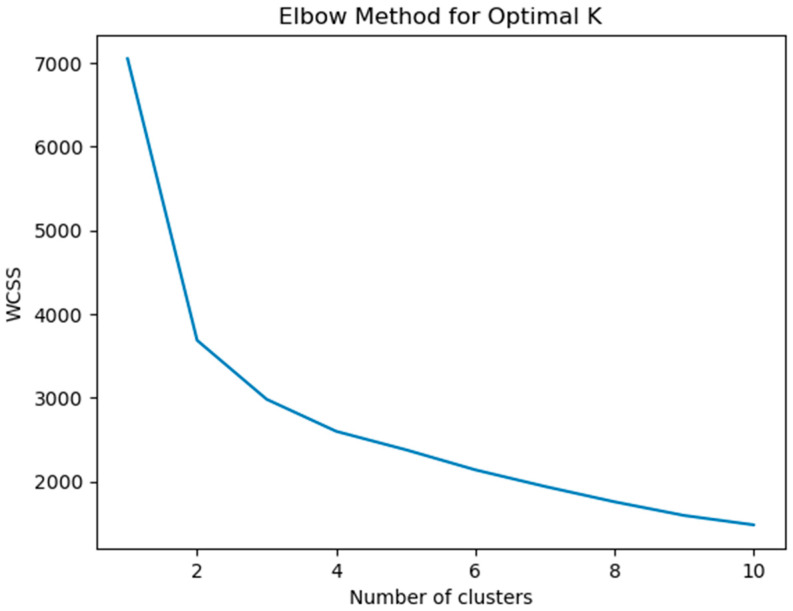
Elbow method for optimal K.

To determine the optimal number of clusters for the K-Means clustering analysis, the Elbow Method was applied. Figure 4 shows the plot of the Within-Cluster Sum of Squares (WCSS) as a function of the number of clusters, ranging from 1 to 10.

The Elbow Method identifies the optimal number of clusters by looking for the point at which the rate of decrease in WCSS slows significantly, forming an “elbow.” In Figure 4, the sharp drop in WCSS from 1 to 2 clusters is followed by a more gradual decline as the number of clusters increases. Although the most significant change occurs between 1 and 2 clusters, the plot indicates that adding more clusters (particularly 4 clusters) still provides meaningful reduction in variance, and the improvement starts to plateau after that.

Therefore, the 4 clusters chosen in the study represent a reasonable balance between minimizing WCSS and ensuring meaningful segmentation. These four clusters correspond to the key speech categories, Atypical, Typical Development (TD), Articulation, and Delay, each of which was identified as an important factor in classifying speech development patterns. Figure 4 demonstrates the trade-off between cluster number and WCSS, helping justify the choice of four clusters as the optimal solution for analyzing speech development trends in this study. The four clusters provide the necessary granularity to distinguish different speech patterns effectively while maintaining model simplicity and interpretability.

**Figure 5:**
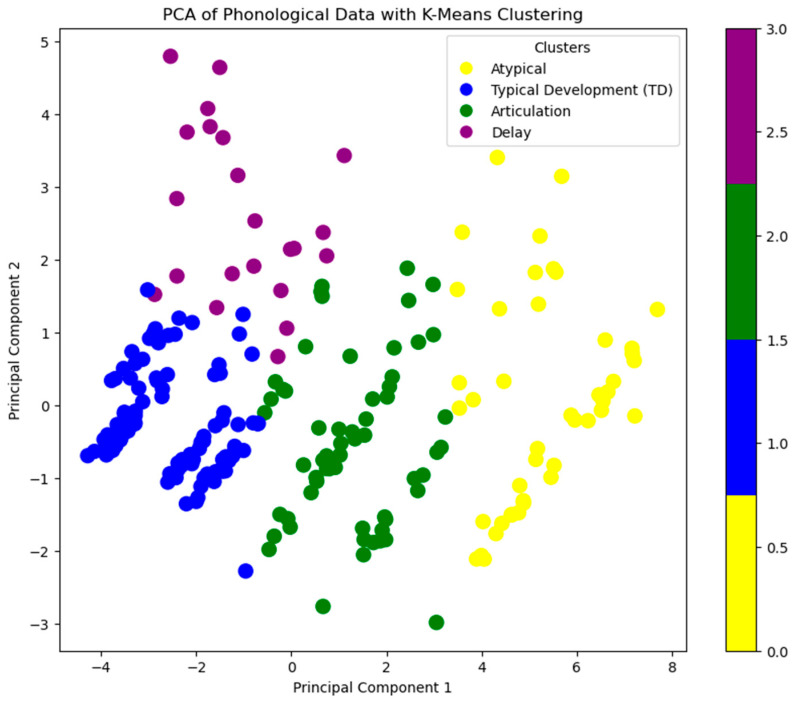
PCA of phonological data with K-Means clustering.

To further explore the data and visualize the distribution of the clusters, Principal Component Analysis (PCA) was applied to reduce the dimensionality of the dataset. Figure 5 shows the 2D representation of the phonological data, with the four identified clusters color-coded: Atypical (yellow), Typical Development (TD) (blue), Articulation (green), and Delay (purple).

The plot effectively captures the variation in speech development across the four clusters. The clusters are well-separated along Principal Component 1 (PC1), with each category clearly grouped based on its phonological features.

Atypical (yellow): This group, representing atypical speech development, is positioned distinctly towards the top-right of the plot, indicating that children in this group show phonological characteristics that are significantly different from the other groups.

Typical Development (TD) (blue): The TD group appears in the lower half of the plot, suggesting that their phonological patterns align with expected developmental milestones for their age.

Articulation (green): This cluster is positioned between the TD and Delay groups, indicating that children in this category exhibit some atypical features, but are closer to the TD group than the Delay group.

Delay (purple): Children in this cluster exhibit the most distinct phonological patterns, with this group being positioned separately from the other three clusters.

The color-coded scatter plot provides an intuitive understanding of how phonological features relate to each group, making it easier to differentiate between typical and atypical speech patterns. The plot illustrates that clustering based on these phonological features effectively identifies distinct subgroups in the dataset, which is valuable for further analysis of speech development in Saudi Arabic-speaking children.

Following the application of PCA for dimensionality reduction and K-Means clustering to identify distinct subgroups based on phonological data, the performance of the machine learning models was re-evaluated. The integration of cluster labels as additional features improved the models’ ability to classify speech development patterns more effectively.

Table 3 shows the updated evaluation results after incorporating the clustering features into the models:

After incorporating the clustering features, both Random Forest and XGBoost demonstrated the best performance, with Accuracy, Precision, Recall, F1-score, and AUC all significantly higher than those of the other models. Both models achieved an AUC greater than 0.98, indicating their strong ability to distinguish between different speech development categories. SVM, while also performing well, fell behind the ensemble methods, with a slightly lower AUC and F1-score. Logistic Regression showed reasonable performance, but displayed a slight drop in metrics compared to the tree-based models. On the other hand, K-Nearest Neighbors (KNN) and Naïve Bayes exhibited the lowest performance, particularly in terms of F1-score and AUC, suggesting that these models had more difficulty in accurately classifying the speech development categories.

Integrating the PCA-derived cluster labels into the models significantly enhanced their ability to differentiate between typical and atypical speech development patterns. Random Forest and XGBoost, both ensemble methods, were the most effective, likely due to their superior capability in handling complex feature interactions and utilizing the additional cluster information. These findings underscore the importance of combining dimensionality reduction and clustering techniques with machine learning models to improve their performance in analyzing speech development.

The clustering results revealed four distinct subgroups based on the speech development patterns observed in the dataset. Typical Development (TD) was the largest subgroup, comprising 110 children, representing 46.61% of the total sample. This group displayed characteristics consistent with typical speech development patterns.

The second-largest subgroup, Articulation Issues, consisted of 60 children, or 25.42% of the sample. These children exhibited speech patterns that indicated specific articulation difficulties, but did not fall into the category of atypical development.

The third group, Atypical Speech Development, included 44 children, accounting for 18.64% of the sample. This subgroup showed significant deviations from typical speech patterns, suggesting atypical developmental trajectories.

Finally, the Speech Delay group, which represented 9.32% of the total sample (22 children), exhibited delays in speech development compared to age-appropriate norms, but did not necessarily show atypical phonological patterns.

These findings, illustrated in Table 4, highlight the heterogeneity in speech development within the study population, with a significant proportion of children exhibiting either articulation issues or delayed speech development. The cluster labels derived from the analysis provide valuable insights into these distinct developmental categories, offering a basis for targeted intervention strategies in speech therapy.

## 4. Discussion

The results of this study offer critical insights into the classification of speech development patterns among monolingual Saudi Arabic-speaking children. By leveraging machine learning models, including Random Forest, XGBoost, SVM, Logistic Regression, K-Nearest Neighbors, and Naïve Bayes, this research aimed to distinguish between four key speech categories: Atypical, Typical Development (TD), Articulation Issues, and Speech Delay. The performance of these models was assessed based on standard metrics, including accuracy, precision, recall, F1-score, and AUC. The Random Forest and XGBoost models emerged as the most effective classifiers, demonstrating their superior capacity to differentiate between these speech categories.

### 4.1. Strengths of Random Forest and XGBoost Models

The remarkable performance of the Random Forest and XGBoost models can be attributed to their ensemble nature, which allows them to combine multiple decision trees to make more robust predictions. These models are particularly adept at capturing complex, non-linear relationships between the features, making them best for this study where phonological features exhibit intricate patterns. Moreover, these algorithms can model interactions between features effectively, which is crucial when working with speech data that involves high-dimensional features such as Phonology Patterns, InfrVar, and PCC.

The significant improvement in model performance after incorporating cluster labels derived from PCA and K-Means clustering further reinforces the importance of this approach. By adding cluster information to the models, we were able to enhance the differentiation between Atypical and TD children, leading to improved accuracy and higher AUC scores. This suggests that the clustering approach helped capture additional structure in the data, allowing the models to classify speech patterns with greater precision.

### 4.2. Support Vector Machine (SVM) Performance

While SVM also performed relatively well, its metrics were slightly lower than those of Random Forest and XGBoost. The AUC and F1-score for SVM were lower, indicating that this model struggled more in distinguishing between some of the subtler differences in speech development patterns. SVM is a powerful algorithm when the data have clear linear decision boundaries, but it may not perform as well when the relationships between features are highly non-linear, as is the case with the phonological features in this study. This may explain why SVM showed slightly reduced performance compared to the tree-based methods.

### 4.3. Logistic Regression and Its Limitations

Logistic Regression exhibited reasonable performance, but showed a noticeable drop in accuracy and AUC compared to Random Forest and XGBoost. Logistic regression is a relatively straightforward model that assumes a linear relationship between the features and the outcome. However, this assumption may not hold true in complex classification tasks, such as those involving speech development, where the relationships between features are often non-linear and multifactorial. Consequently, relying on logistic regression in such contexts may limit the model’s ability to accurately capture the intricate and dynamic nature of speech development. The AUC score of 0.930 suggests that while it provides a decent classification model, it does not capture the complexities of phonological variations as effectively as the ensemble methods. This could be due to its inability to model feature interactions and non-linearities, as well as tree-based algorithms.

### 4.4. K-Nearest Neighbors (KNN) and Naïve Bayes

Both K-Nearest Neighbors (KNN) and Naïve Bayes exhibited the lowest performance among the models tested. KNN, despite being a simple and effective algorithm for some tasks, struggles with high-dimensional data, which often leads to reduced classification accuracy. The F1-score and AUC for KNN were significantly lower, suggesting that it was unable to accurately capture the subtle distinctions between speech development categories. Naïve Bayes, with its assumption of feature independence, is also less suitable for this type of task. Phonological features are likely correlated with each other, and the Naïve Bayes model’s inability to account for these dependencies likely explains its poor performance in this context.

### 4.5. SHAP Analysis: Interpreting Feature Importance

The SHAP (SHapley Additive exPlanations) analysis provided valuable insights into which phonological features were most influential in the classification decisions of the Random Forest, Logistic Regression, and XGBoost models. The Number of Articulation Patterns and Number of Phonology Patterns were identified as the most important features for distinguishing between the Atypical and TD groups, with these features showing the highest impact on model predictions. The prominence of these features aligns with the existing literature, which emphasizes the importance of phonological variations in speech development, especially when identifying atypical patterns.

Other significant features identified in the SHAP analysis, such as PCC (Percentage of Consonants Correct) and InfrVar (Infrequent Variance), further highlighted the critical role of specific phonological measures in distinguishing between Articulation Issues and Speech Delay. The SHAP results confirm that PCC and InfrVar are effective markers for identifying phonological difficulties and delays, with SHAP values ranking these features consistently within the top five across all models. This reinforces their clinical utility, especially when used alongside Articulation and Phonology Pattern counts, which demonstrated the highest SHAP contributions overall. Across all models, the top five features by SHAP importance were (1) Number of Articulation Patterns, (2) Number of Phonology Patterns, (3) Number of Delay Patterns, (4) Percentage of Consonants Correct (PCC), and (5) Infrequent Variance (InfrVar), demonstrating their strong predictive influence on classification decisions

These SHAP-based insights align with our empirical findings regarding Infrequent Variance (InfrVar). In this study, a high incidence of InfrVar was interpreted as a potential red flag for atypical phonological development. While not diagnostic in isolation, elevated InfrVar values, particularly when combined with low PCC or a high number of atypical patterns, served as strong indicators of SSD risk or presence. This rationale is supported by prior phonological research, and our analysis of 235 monolingual Saudi Hejazi Arabic-speaking children further substantiates it [11,12]. InfrVar accounted for a substantial portion of phonological variation, and its progressive reduction with age corresponded with developmental stabilization. This trend reinforces InfrVar’s value as a sensitive diagnostic marker. Unlike earlier studies that often excluded rare phonological errors, our approach integrates InfrVar to model phonological variability that may otherwise be dismissed, thereby enhancing early identification efforts in culturally specific speech assessment.

### 4.6. K-Means Clustering and Subgroup Identification

The K-Means clustering approach identified four distinct subgroups within the dataset, representing different speech development patterns: Typical Development (TD), Articulation Issues, Atypical Speech Development, and Speech Delay. The largest group, TD, comprised 46.6% of the sample, reflecting typical speech development characteristics. Articulation Issues (25.4%) and Atypical Speech Development (18.6%) represented a significant proportion of children, indicating that a large segment of the population exhibited some level of phonological variation that warranted attention. The smallest group, Speech Delay (9.3%), highlighted the presence of children who exhibited delays in speech development, although they did not show atypical phonological patterns.

These results emphasize the heterogeneity of speech development and the need for targeted interventions based on the specific challenges children face. The clustering approach allowed us to uncover subgroups that may have been overlooked using traditional methods, providing a clearer picture of the diverse developmental trajectories in the sample. Although this study focused on Saudi Hejazi Arabic, the classification model was trained on abstracted phonological features rather than raw speech, allowing for potential generalization to other Arabic dialects. Provided that dialect-specific normative data are available and processed using the same analytic framework, the model could be retrained or adapted for broader use across diverse Arabic-speaking populations.

Additionally, this study did not compare the results to non-machine learning methods such as expert-derived scoring or rule-based classification frameworks. This was due to the exploratory nature of the analysis and the focus on evaluating the diagnostic utility of machine learning algorithms. Future research should include comparative benchmarks against traditional clinical or statistical approaches to more comprehensively assess the added value of ML-based tools in speech disorder diagnostics.

The classification criteria employed in this study, specifically the use of a 10% occurrence threshold for phonological pattern classification and the application of the M–1SD cutoff for PCC and other measures, were based on well-established practices in speech-language pathology. These thresholds are commonly used in clinical phonological assessments to differentiate between age-appropriate, delayed, and deviant patterns [30,31,32,33]. For instance, the DEAP diagnostic framework adopts similar thresholds for determining clinical significance [33], while normative studies such as Dodd et al. support the use of frequency-based criteria for distinguishing typical and atypical speech development [30]. Additionally, Shriberg et al. provided empirical validation for the use of PCC and its standard deviation adjustments in identifying children at risk for speech sound disorders [32]. Although the dataset used here was originally normative, our application of these thresholds allowed for meaningful identification of phonological profiles consistent with atypical development. Nonetheless, these thresholds serve as heuristic tools, and future studies should validate their diagnostic sensitivity and specificity against clinical evaluations in diverse populations.

### 4.7. Limitations

This study has several limitations that should be acknowledged. First, the dataset used was originally collected as part of a normative study and did not include formally diagnosed cases of speech sound disorders (SSDs). As such, while the classification models identified patterns consistent with atypical development, these findings require validation against clinically confirmed data. Second, the machine learning models were trained using default hyperparameters without extensive tuning or optimization, which may limit their performance relative to fully optimized models. Third, the speech data were based on structured single-word naming tasks rather than spontaneous speech samples, which may restrict the generalizability of the findings to naturalistic settings.

Fourth, while the study focused on Saudi Hejazi Arabic, broader generalization to other dialects of Arabic would require retraining the models using dialect-specific phonological norms. Fifth, the use of principal component analysis (PCA) prior to K-Means clustering, while effective for linear dimensionality reduction, may not capture non-linear relationships in the data as well as alternatives like t-SNE or UMAP. Sixth, some diagnostic subgroups, such as Atypical (*n* = 22) and Delay (*n* = 37), were underrepresented compared to others, which may reduce the statistical power and generalizability of findings for these categories. Seventh, model performance was evaluated only on internal train–test splits; no external validation dataset was used, which may limit generalizability across populations or settings.

Eighth, although phonological transcriptions were performed by a certified speech-language pathologist with high inter- and intra-rater reliability, manual transcription inherently carries the risk of subjectivity and perceptual bias. Future studies may benefit from incorporating semi-automated transcription tools to enhance objectivity and reproducibility. Finally, the study design was cross-sectional, limiting the ability to evaluate the longitudinal predictive validity of measures like InfrVar. Prospective, longitudinal studies are needed to determine whether these phonological markers reliably predict persistent SSD outcomes over time.

Future work should address these limitations by including clinical samples, conducting hyperparameter optimization, incorporating spontaneous speech data, applying advanced dimensionality reduction techniques, expanding to additional dialects, validating models with external datasets, and exploring longitudinal developmental trajectories.

## 5. Conclusions

In conclusion, the combination of machine learning models, dimensionality reduction (PCA), and clustering techniques has proven to be effective in analyzing speech development in Saudi Arabic-speaking children. The Random Forest and XGBoost models performed exceptionally well, with AUC scores above 0.98, indicating their high capacity to distinguish between different speech development patterns. The integration of cluster labels derived from PCA and K-Means clustering further improved the models’ performance, highlighting the value of clustering in capturing latent patterns in the data. Additionally, the SHAP analysis provided critical insights into the key phonological features, such as Number of Articulation Patterns and PCC, which were instrumental in classifying speech development categories. These findings underscore the importance of using advanced machine learning techniques to analyze and interpret complex speech data, providing a foundation for more targeted and effective interventions for children with speech delays or disorders.

## Figures and Tables

**Table 1:** Participant distribution by age group and gender.

Age Groups (Year; Month)	*N*	Female	Male	Mean Age (Months)	SD Age (Months)	% of Sample
2; 6–2; 11	30	15	15	32.20	1.86	12.77
3; 0–3; 5	27	14	13	30.00	1.92	11.49
3; 6–3; 11	38	17	21	45.24	1.79	16.17
4; 0–4; 5	39	17	22	50.33	1.63	16.60
4; 6–4; 11	31	16	15	57.19	1.74	13.19
5; 0–5; 5	37	22	15	62.38	1.53	15.74
5; 6–5; 11	33	18	15	68.45	1.79	14.04
Total	235	119	116			

Note: Participants were stratified into 6-month age bands to allow age-specific comparisons of phonological measures.

**Table 2:** Model evaluation results.

Model	Accuracy	Precision	Recall	F1-Score	AUC
XGBoost	91.49%	92.73%	91.49%	91.32%	99.14%
Random Forest	91.49%	91.71%	91.49%	91.38%	98.17%
Logistic Regression	89.36%	90.66%	89.36%	89.31%	98.44%
SVM	85.11%	89.64%	85.11%	84.76%	98.70%
K-Nearest Neighbors	85.11%	86.10%	85.11%	85.06%	95.47%
Naïve Bayes	55.32%	41.09%	55.32%	44.46%	90.23%

**Table 3:** Updated models evaluation.

Model	Accuracy	Precision	Recall	F1-Score	AUC
Random Forest	93.62%	93.88%	93.62%	93.57%	98.80%
XGBoost	93.62%	93.88%	93.62%	93.57%	98.63%
SVM	86.17%	86.69%	86.17%	86.14%	95.40%
Logistic Regression	85.11%	85.11%	85.11%	85.06%	93.06%
K-Nearest Neighbors	78.72%	79.03%	78.72%	78.57%	91.94%
Naïve Bayes	59.57%	69.04%	59.57%	52.48%	91.39%

**Table 4:** Distribution of Speech Development Subgroups Identified Through Clustering.

Cluster	Subgroup Name	Number of Children	Percentage%
0	Atypical Speech Development	44	18.64
1	Typical Development (TD)	110	46.61
2	Articulation Issues	60	25.42
3	Speech Delay	22	9.32

## Data Availability

Data supporting the results reported in this study are available upon reasonable request. Due to privacy and ethical considerations, the dataset generated and analyze during this study is not publicly available. Requests for access to the data can be directed to the corresponding author and will be evaluated in accordance with institutional and ethical guidelines.
